# Metal-Assisted Hydrolysis Reactions Involving Lipids: A Review

**DOI:** 10.3389/fchem.2019.00014

**Published:** 2019-02-19

**Authors:** Dominique E. Williams, Kathryn B. Grant

**Affiliations:** ^1^Department of Chemistry, University of Richmond, Richmond, VA, United States; ^2^Department of Chemistry, Georgia State University, Atlanta, GA, United States

**Keywords:** cleavage, fatty acids, liposomes, phospholipase mimics, triglycerides

## Abstract

This report covers major advances in the use of metal ions and complexes to hydrolyze ester and phosphate ester lipid bonds. These metal-based Lewis acids have been investigated as catalysts to isolate fatty acids from biological sources, as probes to study phospholipid bilayer properties, as tools to examine signal transduction pathways, and as lead compounds toward the discovery of therapeutic agents. Metal ions that accelerate phosphate ester hydrolysis under mild conditions of temperature and pH may have the potential to mimic phospholipase activity in biochemical applications.

## Introduction

**Figure 1:**
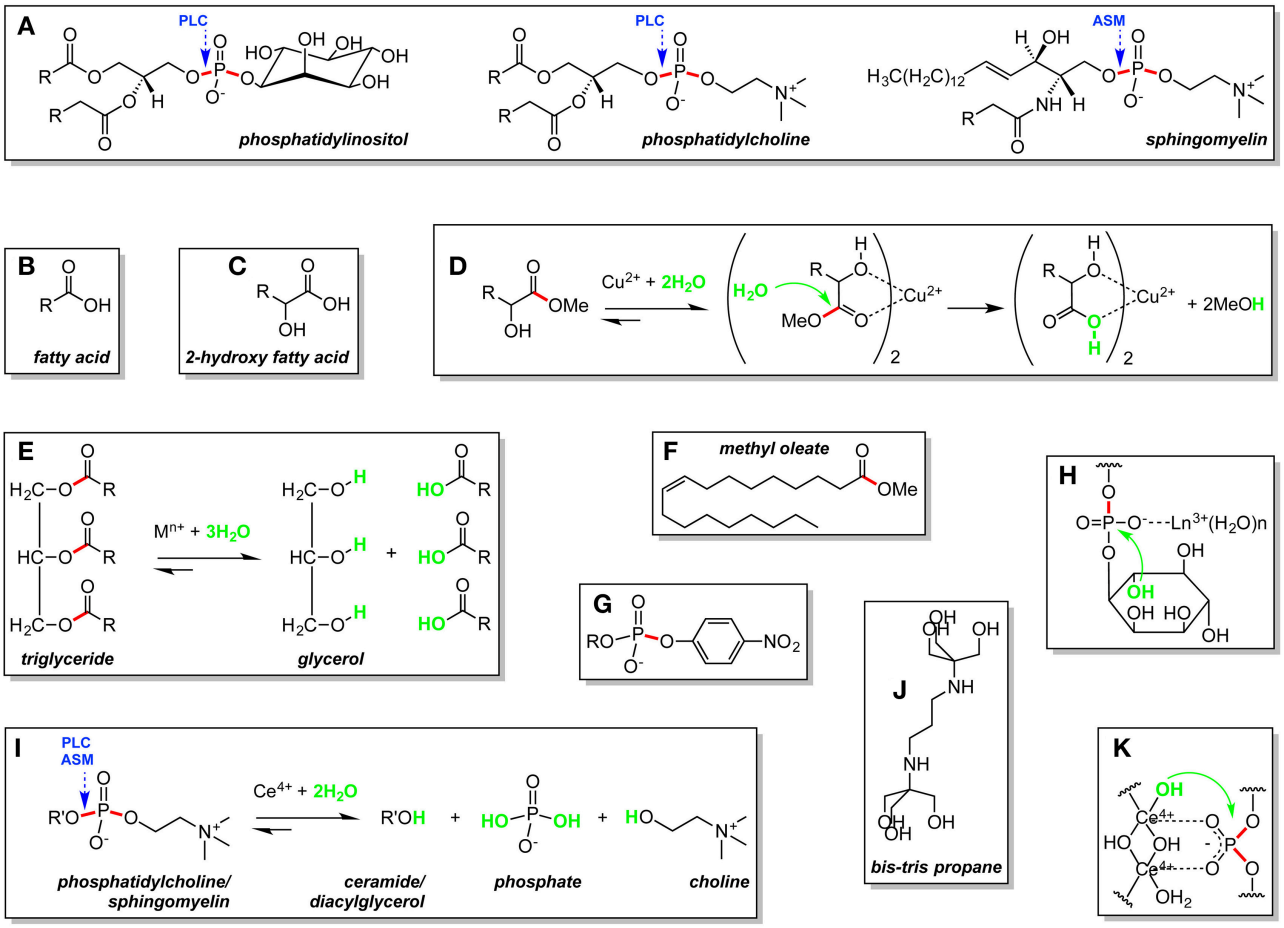
Lipid **(A–G)** and ligand **(J)** structures, metal-assisted lipid hydrolysis reactions **(D,E,I)**, and metal-assisted lipid hydrolysis mechanisms **(D,H,K)** described in the manuscript. R, hydrocarbon chain; R', diacylglycerol for phosphatidylcholine and ceramide for sphingomyelin; M, metal; n+, charge. Scissile ester bonds are in red. Nucleophiles are in green. Bond hydrolyzed by phospholipase C (PLC) and acid sphingomyelinase (ASM) are identified with blue arrows.

Metal ions and complexes that hydrolyze biological molecules have become increasingly important to the fields of chemistry and biology (Grant and Kassai, 2006; Mancin et al., 2012, 2016; Wezynfeld et al., 2016; Yu et al., 2016). The majority of the studies in this area have focused on the reversible addition of water across ribo- and deoxyribonucleic acid phosphodiester bonds and peptide and protein amide bonds. Hydrolytically active metal ion centers such as Ce(IV), Co(II), Co(III), Cu(II), Fe(III), Ln(III), Ni(II), Mo(IV), Pd(II), Zn(II), and Zr(IV) have been considered for a number of diverse applications, e.g., as probes to study protein function and solution structure, as enzyme models that examine metallo-hydrolase activity, and as hydrolytic agents in nucleic acid and protein engineering experiments. Although metal-assisted hydrolysis of lipids remains relatively unexplored, it is undoubtedly of equal importance. Lipids play central roles in biological systems as energy-storage molecules and as chemical messengers in cell signaling (Wenk, 2010). As the major components of the biological membranes that surround all cells and organelles, phospholipids are of particular significance to almost all known life forms (Figure 1A).

## Isolation and Recycling of Fatty Acids From Biological Sources

Fatty acids (Figure 1B) are key building blocks of complex lipid molecules including phospholipids, triglycerides, and sterol esters. Lipids containing 2-hydroxy fatty acid units (Figure 1C) are found in wool wax, microorganisms, as well as in the animal central nervous system, skin, and kidney (Kishimoto and Radin, 1963). In phospholipid bilayers, the 2′-OH group of 2-hydroxy lipids forms hydrogen bonds that strengthen membrane structure (Hama, 2010).

In one of the earliest reports appearing on metal-assisted lipid hydrolysis, Wernette and co-workers isolated free 2-hydroxy fatty acids by using Cu(NO_3_)_2_ to hydrolyze 2-hydroxy fatty acid methyl esters (50°C in water or methanol-water, 3 to 6 h) (Boyer et al., 1979). Methyl ester cleavage was proposed to occur via the formation of a bis complex containing a central copper(II) ion and two 2-hydroxy fatty acid ligands (Figure 1D). Interaction of the metal ion center of the complex with the methyl ester carbonyl oxygen atom of each fatty acid unit activated the corresponding carbonyl carbon toward nucleophilic attack by water (or hydroxide), leading to the production of two equiv. of methanol and a Cu(2-hydroxy acid)_2_ precipitate. Subsequent EDTA treatment released the 2-hydroxy fatty acid ligands in 80–82% yield (Entry 1 in Table S1, Supplementary Material). Wernette suggested that it should be possible to employ Cu(II) ions to hydrolyze complex lipids present in biological extracts, allowing for naturally occurring 2-hydroxy fatty acids to be isolated readily.

**Graphical Abstract:**
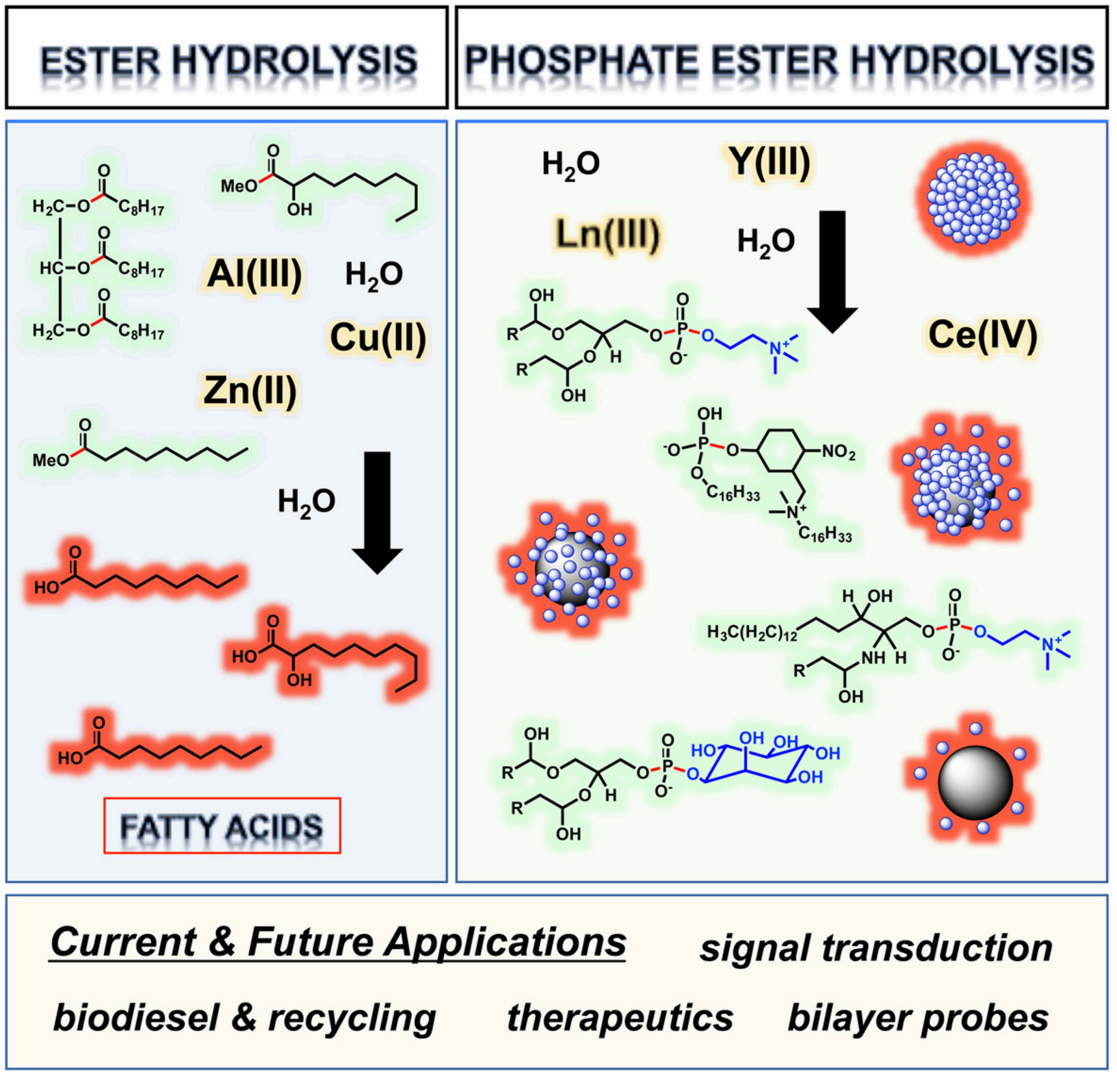
Metal ion centers hydrolyzing ester bonds in lipids and the phosphate ester bonds of phospholipid liposomes, with scissile linkages and polar head groups respectively highlighted in red and blue.

Free fatty acid building blocks have also been generated by using metal ions to hydrolyze triglycerides (Figure 1E). In addition to their importance as key components of biological lipids, fatty acids are of considerable commercial interest as a raw material in the manufacture of detergents, soaps, lubricants, plasticizers, and biodiesel, a fuel consisting of mono alkyl esters prepared using fatty acids from vegetable oil and animal fat (Knothe, 2010). Recycling of fatty acids from *waste oils* has led to important new, environmentally friendly processes for biodiesel production (Hajjari et al., 2017). Toward these ends, Ratnasamy et al. reacted the solid Fe(II)-Zn(II) double-metal cyanine catalyst K_4_Zn_4_[Fe(CN)_6_]_3_•H_2_O (Fe-Zn DMC) with vegetable oils and animal fats in batch reactors (Satyarthi et al., 2011). DMC catalysts have zeolite-like cage structures and are traditionally used in the manufacture of polyether polyols (Almora-Barrios et al., 2015). Compared to the 14% hydrolysis yield obtained in the absence of catalyst, Ratnasamy et al. used Fe-Zn DMC to convert triglycerides in the starting material to free fatty acids with a yield of 72% and a turnover number of 25 (190°C in water, 8 h; Entry 2 in Table S1). Tetrahedrally coordinated Zn(II) Lewis acid centers within the catalyst were proposed to activate acyl ester bonds in the triglycerides toward nucleophilic attack by water.

As an innovative approach to biodiesel production, Ismadji et al. utilized synthetic *Cu(II)-laden wastewater* to hydrolyze acylglycerides in waste cooking oil (Ong et al., 2016, 2017). The authors viewed the proposed method as a process that would not only furnish free fatty acid, but one that would remediate wastewater toxicity and conserve the large amounts of water consumed in traditional oil splitting. Using an aqueous solution of CuSO_4_ and waste cooking oil from a local restaurant, a total of 83.0% of the acylglycerides in the oil was hydrolyzed to fatty acids (77.6% yield, 225°C, 8 h; Entry 3 in Table S1) (Ong et al., 2016). While these values were only slightly lower in reactions run with copper-free water (75.7% acylglyceride conversion, 69.6% fatty acid yield), 51.8% of the copper(II) in the wastewater was successfully removed by the hydrolyzed oil phase of the reaction. Cumulative copper(II) detoxification was increased from 51.8 to 85.2% when the same sample of wastewater was utilized to treat a second batch of cooking oil (77.6% acylglyceride conversion, 72.2% fatty acid yield).

While the global production and consumption of biodiesel have expanded, there are concerns that the oxygen atoms present in biodiesel lipids adversely affect stability, energy density, and other fuel properties (Knothe, 2010). Triglycerides from vegetable oils and animal fats have therefore been subjected to direct hydrodeoxygenation reactions to generate oxygen-free, hydrocarbon-based renewable (green) diesel fuels (Knothe, 2010). Coumans and Hensen recently studied the interactions between the heterogeneous sulfided green diesel catalyst NiMo/γ-Al_2_O_3_ and the “model triglyceride” methyl oleate (Figure 1F) (Coumans and Hensen, 2017). The NiMo/γ-Al_2_O_3_ catalyst was prepared by grinding and sieving a porous γ-alumina (γ-Al_2_O_3_) solid support pre-treated with Ni(NO_3_)_2_•6H_2_O and (NH_4_)_6_Mo_7_O_24_•7H_2_O. Reactions between methyl oleate and the catalyst were conducted in a single-pass micro flow reactor under trickle flow conditions (260°C in tetralin, 60 bar, ~2 h; Entry 4 in Table S1). Near-complete conversion of methyl oleate to C17 and C18 hydrocarbons was observed. Based on the distribution of reaction intermediates and products, Coumans and Hensen proposed a reaction pathway in which hydrolysis of methyl oleate to fatty acid intermediates was catalyzed by coordinately unsaturated Al(III) centers of high Lewis acid strength located on the γ-alumina surface (Wischert et al., 2014). Direct hydrodeoxygenation of the fatty acids by the Ni(II)/Mo(VI) metal sulfide phase of the catalyst then gave rise to C18 hydrocarbons, while H_2_S-assisted decarbonylation (or decarboxylation) yielded C17 hydrocarbons.

## Synthetic Lipid Analogs: Analysis of Bilayer Permeability and Dynamics

**Figure 2:**
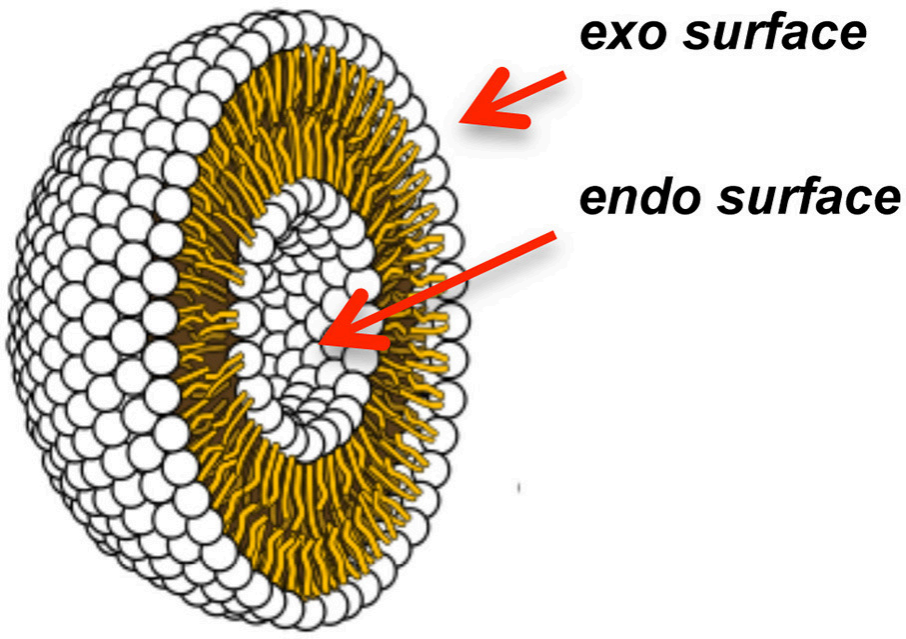
Cross-section through a unilamellar liposome.

Metal ions and complexes that hydrolyze lipids under non-denaturing conditions of temperature and pH have been used to investigate the properties of phospholipid bilayers under physiological conditions (Mancin et al., 2016). In these studies, Moss et al. modeled bilayers by incorporating double-chain synthetic lipid analogs containing *p-nitrophenol activated* phosphate ester bonds into unilamellar liposomes (Figure 2). While *p*-nitrophenol is an excellent colorimetric tool, integrating this chromophore into a synthetic phospholipid (Figure 1G) increases the susceptibility of scissile bonds toward hydrolysis. The liposomes were reacted under mild conditions upon the addition of the lanthanide metal ions Eu(III), Lu(III), Tb(III), Tm(III), and/or Yb(III) to the external bulk solution (25–27°C and pH 7.0–7.3; Entries 5 and 6 in Table S1) (Moss et al., 1995; Scrimin et al., 1998, 2000). Reactions were monitored via colorimetric detection of the *p*-nitrophenolate anion that was released upon metal-assisted hydrolysis (Figure S1). Relevant information relating to transverse lipid diffusion (flip-flop) rates was then revealed. At temperatures below the phase transition temperature (*T*_c_) of the liposome, hydrolysis of the *p*-nitrophenol activated phosphate ester lipid bonds occurred only at the exoliposomal bilayer surface facing the bulk solution, independent of the charge, positive or negative, of the bilayer lipids (Figure 2). This confirmed that free ions are generally unable to permeate across biological membranes. Above the *T*_c_ however, rapid transverse diffusion of the synthetic lipids from the interior endo surface to the exo surface of the liposomes gave rise to additional metal-assisted cleavage. The hydrolytically active lanthanides were also used to manipulate bilayer properties. For example, Moss et al. showed that the permeability barrier of liposomes could be overcome by utilizing lipophilic amine ligands to coordinate to metal ions and then transport them by transverse diffusion from the outer to the inner membrane leaflet (Scrimin et al., 1998). When Ln(III) ions were employed to hydrolyze one of the two aliphatic chains of *p*-nitrophenol activated exo surface lipids (Figure S2), the rapid exposure of the endoliposomal surface to metal ions triggered an escalation in cleavage accompanied by the release of fluorescent reporter molecules stored within the liposomes' interior (Scrimin et al., 2000).

## Metal Ions as Phospholipase Mimics

In addition to synthetically activated phosphate esters, lanthanide ions can hydrolyze unactivated phosphate ester bonds in naturally occurring phospholipids. As strong Lewis acids equipped with high charge densities, high coordination numbers, and rapid ligand exchange rates, the lanthanides are ideally suited as oxophilic, hydrolytic agents (Franklin, 2001). Consistent with the electrostatic nature of lanthanide-ligand interactions, these metal ion centers are drawn to negatively charged oxygen atoms in phospholipid phosphate ester bonds (Hauser and Phillips, 1976). When hydrolysis of unactivated phospholipids occurs at physiological temperature and pH, the lanthanide ions have the potential to mimic phospholipase activity.

Phospholipases are enzymes that hydrolyze ester or phosphate ester bonds. They can be either cytoplasmic or lysosomal in origin. An important example of a cytoplasmic phospholipase is phosphatidylinositol-specific phospholipase C (PLC), which converts the phosphoglyceride phosphatidylinositol (PI; Figure 1A) to diacylglycerol and phosphorylated inositol, important secondary messengers in signal transduction (Figure S3) (Cocco et al., 2015). The lysosome is a cellular organelle that contains acid hydrolases that hydrolytically breakdown macromolecules into their original, monomeric building blocks (Appelqvist et al., 2013). Unlike cytoplasmic enzymes, acid hydrolase activity is typically optimal at lysosomal pH (~ pH 4.8) and significantly lower at cytoplasmic pH (~ 7.2). Examples of acid hydrolases that engage in phosphate ester bond hydrolysis include: (i) acid sphingomyelinase (ASM) (Jenkins et al., 2009), which acts on the sphingolipid sphingomyelin (SM) to release ceramide and (ii) lysosomal phospholipase C (Matsuzawa and Hostetler, 1980), that hydrolyzes phosphoglycerides such as phosphatidylcholine (PC) and phosphatidylinositol to form diacylglycerol (Figure 1A).

### Cytoplasmic Phospholipase Mimics: Tools to Study Signal Transduction

The first phospholipase mimics were discovered by the research groups of Komiyama and Liu, who used Ce(III), Eu(III), La(III), Tb(III), Tm(III), and Y(III) metal ion salts under near-physiological conditions (30–37°C, pH 7.5–8.5; Entries 7 and 8 in Table S1) to hydrolyze naturally occurring, unactivated phosphatidylinositol (Figure 1A) in liposomes (Matsumura and Komiyama, 1994) and intact erythrocyte membranes (Liu et al., 2001). The rare earth ions “mimicked” the activity of cytoplasmic phosphatidylinositol-specific phospholipase C by converting the phosphatidylinositol to diacylglycerol and phosphorylated inositol (Figure S3). The two most active metal ions were Y(III) in PI liposomes (32% yield, 30°C and pH 8.0, 24 h) and La(III) in PI-laden erythrocyte membranes. In contrast, hydrolysis was not observed when PC liposomes were treated with Y(III). Komiyama thus proposed a hydrolytic mechanism in which a rare earth ion binds to a negatively charged phosphate oxygen atom of PI, activating the phosphorous atom toward nucleophilic attack by the 2-hydroxy group specific to inositol (Figure 1H). The results of these investigations suggested that it should be possible to use hydrolytically active rare earth metal ions as “cytoplasmic phospholipase mimics” to study signal transduction pathways, e.g., via the generation of diacylglycerol and phosphorylated inositol in phospholipase deficient cell lines and animal models (Li et al., 2000).

### Mimicking Lysosomal Phospholipase: A Potential Therapeutic Application for Cerium(IV)

Williams, Grant et al. have focused on phosphatidylcholine (Figure 1A) and sphingomyelin (Figure 1A) (Kassai et al., 2011; Cepeda et al., 2012), which make up approximately 50% of the phospholipid content of eukaryotic bilayer membranes. The authors utilized unactivated liposomes to model biological membranes and phosphate-specific colorimetric detection based on malachite green to quantitate hydrolysis. When PC and SM liposomes were treated with metal ion salts of Ce(IV), Zr(IV), Hf(IV), Co(II), Cu(II), Eu(III), La(III), Ni(II), Pd(II), Y(III), Yb(III), and Zn(II) at 60°C (20 h), cerium(IV) displayed overwhelmingly superior levels of phosphodiester cleavage, releasing inorganic phosphate in appreciable yields at lysosomal pH (~4.8; PC 41%, SM 22%) and in low yields under near neutral conditions (~pH 7.2; PC 13%, SM 5%; Entries 9 and 10 in Table S1; Figure 1I). Two major factors where proposed to account for the preference of Ce(IV) for mildly acidic conditions. The p*K*_a_ value of Ce(IV)-bound water is approximately −1.1 (Wulfsberg, 1987), which enables this metal ion center to generate hydrolytically active hydroxide nucleophiles even at low pH values. Secondly, the multinuclear Ce(IV) hydroxo species responsible for phosphodiester hydrolysis lose positive charge and Lewis acid strength as reaction pH is raised (Maldonado and Yatsimirsky, 2005). Among the lanthanide(III) ions tested, liposomes were cleaved only at pH 7.2, albeit in extremely low yields < 2%. Unlike cerium(IV), water molecules bound to Ln(III) ions have p*K*_a_ values that support phosphate ester bond hydrolysis under neutral to slightly alkaline conditions, e.g., ~8.0 for Eu(III), ~8.0 for Yb(III), and ~8.5 for La(III) (Burgess, 1978; Wulfsberg, 1987). The hydrolytic superiority of cerium is likely to be related to its +4 oxidation state (Bracken et al., 1997). In addition to increasing the acidity of metal-bound water, the elevated charge density of Ce(IV) intensifies its Lewis acid strength.

The high cerium(IV) activity at lysosomal pH coupled with the ability to hydrolyze SM and PC phosphate ester bonds on the ceramide/diacylglycerol side of phosphate suggest that this metal ion center might serve as an acid sphingomyelinase or lysosomal phospholipase C mimic (Figure 1I). In order for such an enzyme mimic to be optimal, cleavage should occur at physiological temperature and should be greatly diminished in neutral environments. To further enhance and tune cerium(IV) chemistry, Williams et al. turned to the chelating ligand bis-tris propane (BTP; Figure 1J) (Williams et al., 2015). Upon the addition of BTP to optimized 37°C reactions, cerium(IV) hydrolyzed unactivated PC liposomes to release 5.7 times more inorganic phosphate at ~pH 4.8 than at ~pH 7.2, a major enhancement compared to the ~2.1 fold increase that was observed in ligand free controls (20 h). In the presence of BTP, the yield of inorganic phosphate at pH 4.8 and 37°C was 67%, a value that is roughly equivalent to the percent of phospholipid molecules found on the metal-accessible exo surface of small liposomes (Entry 11 in Table S1). NMR studies indicated that the pH-dependent “on-off switch” of the BTP ligand is related to the p*K*_a_ values of its nitrogen donor atoms (p*K*_a1_ = 6.8, p*K*_a2_ = 9.1) (Maldonado and Yatsimirsky, 2005; Williams et al., 2015). At pH 4.8, near-complete donor atom protonation minimizes interactions between cerium(IV) and BTP. The hydrolytically active multinuclear Ce(IV) hydroxo species are unhindered and free to promote hydrolysis through a mechanism in which Ce(IV) binds to negatively charged phosphate oxygen atoms in the lipid, activating phosphorous toward attack, while delivering a hydroxide nucleophile to an adjacent phosphodiester bond (Figure 1K) (Komiyama et al., 1999). At pH 7.2, nitrogen deprotonation enables BTP to bind to Ce(IV) and impede its ability to accelerate cleavage. The promising *in vitro* results pointing to cerium(IV) as a “lysosomal phospholipase mimic” are consistent with a small molecule approach to reversing the pathogenic lysosomal build-up of sphingomyelin that occurs in Niemann-Pick disease type A, a fatal lysosomal storage disease caused by mutations in the human ASM gene (Schuchman and Desnick, 2017).

In a related study, König and co-workers explored the effects of phosphatidylcholine liposomes on the interactions between the activated DNA model compound bis-4-nitrophenyl phosphate (BNPP) and Fe(III), Cu(II), Zn(II), Al(III), La(III), Ce(III), Eu(III), Tb(III), and Yb(III) (25°C and pH 7.4, 24 h; Entry 12 in Table S1; Figure S4) (Poznik et al., 2015). While the *d-* and *p-* block metal ions were less active, the five Ln(III) metal centers accelerated hydrolysis of the phosphodiester bonds of the external BNPP substrate. When unactivated PC liposomes were added to the reactions, the hydrolytic activity of only the lanthanide ions toward BNPP was markedly increased (~11–19% final yield). The lanthanides were then shown to quench the fluorescence of membrane embedded carboxyfluorescein, leading the authors to propose a hydrolytic mechanism in which densely packed Ln(III) Lewis acid centers assembled at the lipid-water interface of the PC liposomes accelerate phosphodiester hydrolysis in a cooperative fashion. Dissimilar to Williams et al.'s data (Kassai et al., 2011; Williams et al., 2015), metal-assisted cleavage of unactivated bilayer phosphatidylcholine molecules was not reported.

## Concluding Remarks

In this review, we have summarized and commented on key research studies in which metal ions and complexes were used to hydrolyze ester and phosphate ester lipid bonds. We found that the metal ion centers Al(III), Cu(II), and Zn(II) cleave neutral, unactivated ester bonds in acylglycerides and fatty acid esters, mainly at elevated temperatures (50–260°C). In contrast, the lanthanide/rare earth ions Ce(III), Eu(III), La(III), Lu(III), Tb(III), Tm(III), Yb(III), Y(III), and Ce(IV) work well at 25–37°C with lipids containing negatively charged phosphate ester bonds. These mild temperature conditions enable phospholipids in fully assembled liposomes to be cleaved. Among the lanthanides, Ln(III) ions were primarily used to hydrolyze *p*-nitrophenol activated phosphate ester lipid bonds in neutral to mildly alkaline pH environments (pH 7.0–8.5). In the case of phosphatidylinositol, the 2-hydroxyl group of inositol serves as an internal nucleophile, permitting Ln(III)-assisted hydrolysis of liposomes to proceed in the absence of a phosphate ester activating group. The lanthanide ion Ce(IV) favors mildly acidic conditions over near neutral pH, and at 37°C is highly reactive toward the hydrolytic cleavage of unactivated phosphatidylcholine phosphate ester bonds. In addition to relating the mechanistic aspects of lipid hydrolysis, the research articles showcased in this review underscore the potential of metal ions and complexes to serve as hydrolytic agents in diverse applications ranging from biofuel production to therapeutics.

## Author Contributions

All authors listed have made a substantial, direct and intellectual contribution to the work, and approved it for publication.

### Conflict of Interest Statement

The authors declare that the research was conducted in the absence of any commercial or financial relationships that could be construed as a potential conflict of interest.
