# Supplementary material for: Metal-Assisted Hydrolysis Reactions Involving Lipids: A Review
Source: Front Chem. 2019 Feb 19;7:14. doi: 10.3389/fchem.2019.00014 (PMC6390409; doi:10.3389/fchem.2019.00014)
Supplement: Supplementary file 1 [file Data_Sheet_1.pdf]

Supplementary Material

Metal-Assisted Hydrolysis Reactions Involving Lipids: A Review

Dominique E. Williams[1] and Kathryn B. Grant[2*]

[1] Department of Chemistry, University of Richmond, Richmond, VA USA

[2]Department of Chemistry, Georgia State University, Atlanta, GA USA

** Corresponding Author: kbgrant@gsu.edu*

TABLE S1 | Metal-assisted hydrolysis reactions involving lipids[a]

**Hydrolytically active agent(s)**	**Substrate(s)**	**Product(s) detected**	**Solvent, pH**	**Temp (°C)**	**Time (h)**	**Yield (%)**	**Notes**	**Ref.**
**1:** $Cu(NO_3)_2$	2-hydroxy fatty acid methyl esters	2-OH fatty acids	H_2O, MeOH	50	3-6	80-82	2-hydroxy fatty acids hydrolysis products recovered using EDTA	[1]
**2:** solid $K_4Zn_4[Fe(CN)_6]_3$ •H_2O DMC catalyst	triglycerides in soybean oil	fatty acids	H_2O	190	8	72	active Lewis acid center = Zn(II); TON = 25; hydrolysis yield = 14% without catalyst	[2]
**3:** $CuSO_4$-laden synthetic wastewater	acylglycerides in waste cooking oil	fatty acids	H_2O	225	8	78	52% of Cu(II) removed from wastewater by hydrolyzed oil phase; hydrolysis yield = 70% without catalyst	[3]
**4:** solid sulfided NiMo/γ-Al_2O_3 catalyst	methyl oleate	fatty acids (inferred)	tetralin	260	2	~complete	pressure = 60 bar; active Lewis acid center = Al(III)	[4]
**5:** $EuCl_3$, $LuCl_3$, $TbCl_3$, $TmCl_3$, $YbCl_3$	1,2-dipalmitoyl-3-glyceryl *PNP* phosphate liposomes	*PNPO*	pH 7.0-7.3	25-27	n.r.	50-70	liposomal bilayer impermeable to Ln(III): hydrolytic *PNPO* release was quantitative and occurred only on exolipsomal surface; exoliposomal specificity disappeared above T_c due to lipid "flip-flop"; metal-chelating lipophilic amines transported Ln(III) across bilayer	[5, 6]
**6:** $EuCl_3$, $TbCl_3$, $TmCl_3$,	double-chain hexadecyl di-methylammonium methyl *PNP* phosphate liposomes	single-chain hexadecyl *PNPO*	pH 7.0	25	n.r.	~complete	Ln(III)-assisted hydrolysis released an internal fluorescent reporter, exposed endoliposomal lipids to metal ions, and increased hydrolysis yields	[7]
**7:** $CeCl_3$, $EuCl_3$, $LaCl_3$, $TmCl_3$, YCl_3	PI liposomes	DAG	pH 8.0	30	24	9-32	YCl_3: highest activity; $CuCl_2$, $FeCl_3$, $ZnCl_2$: totally inactive; PC liposomes were not hydrolyzed by YCl_3	[8]
**8:** $CeCl_3$, $LaCl_3$, $TbCl_3$, YCl_3	PI in erythrocyte membranes	DAG, IP_3	pH 7.8-8.0	37	24-30	n.r.	$CeCl_3$ and $LaCl_3$: highest activity	[9]
**9:** $Ce(NH_4)_2(NO_3)_6$	PC liposomes	P_i	pH 4.8	60	20	41	$ZrCl_4$, $HfCl_4$: extremely low activity; $CoCl_2$, $CuCl_2$, $EuCl_3$, $LaCl_3$, $NiCl_2$, K_2PdCl_4, YCl_3, $YbCl_3$, $ZnCl_2$: totally inactive	[10]
**10:** $Ce(NH_4)_2(NO_3)_6$	SM liposomes	P_i	pH 4.8	60	20	22	$ZrCl_4$, $HfCl_4$, $CoCl_2$, $CuCl_2$, $EuCl_3$, $LaCl_3$, $NiCl_2$, K_2PdCl_4, YCl_3, $YbCl_3$, and $ZnCl_2$: totally inactive	[11]
**11:** $Ce(NH_4)_2(NO_3)_6$ with bis-tris propane	PC liposomes	P_i	pH 4.8	37	20	67	67% ~ equivalent to total phosphate on the exoliposomal surface of small liposomes	[12]
**12:** $CeCl_3$, $EuCl_3$, $La(NO)_3$, $TbCl_3$, & $YbAc_3$-coated PC liposomes	BNPP	*PNPO*	pH 7.4	25	24	~11-19	$Al(NO)_3$, $CuSO_4$, $FeCl_3$, $ZnCl_2$-coated PC liposomes: extremely low BNPP activity; PC in liposomes was not hydrolyzed	[13]

[a] Abbreviations: BNPP, bis-4-nitrophenyl phosphate; DAG, diacylglycerol; DMC, double-metal cyanide; P_i, inorganic phosphate; IP_3, inositol 1, 4, 5-triphosphate; n.r., not reported; PC, phosphatidylcholine; PI, phosphatidylinosiltol; PNP, *p*-nitrophenyl; PNPO, p-nitrophenolate; SM, sphingomyelin; T_c, liposomal phase transition temperature; TON, turnover number

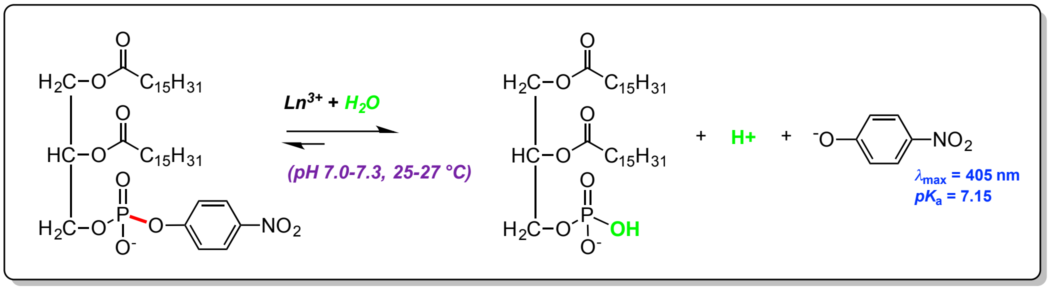

Figure S1. Hydrolytic release of *p*-nitrophenolate anion observed by Moss, Scrimin and co-workers upon reacting 1,2-dipalmitoyl-3-glyceryl *p*-nitrophenyl phosphate liposomes with $EuCl_3$, $LuCl_3$, $TbCl_3$, $TmCl_3$, and $YbCl_3$ (Entry 5 in **Table S1**).[5, 6] Scissile ester bond is in red.

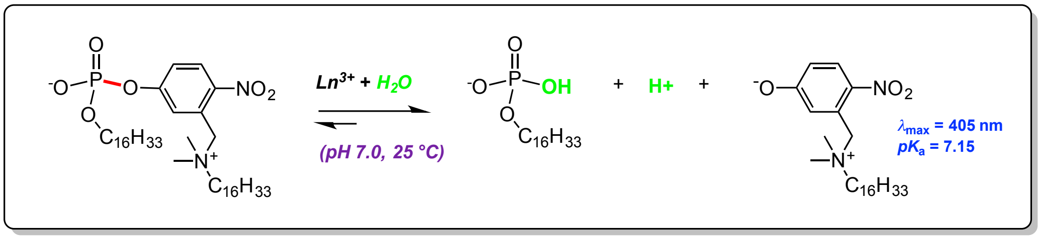

Figure S2. Hydrolytic release of *p*-nitrophenolate anion observed by Scrimin and co-workers upon reacting double-chain hexadecyl dimethylammonium methyl *p*-nitrophenyl phosphate liposomes with $EuCl_3$, $TbCl_3$, and $TmCl_3$ (Entry 6 in **Table S1**).[7] Scissile ester bond is in red.

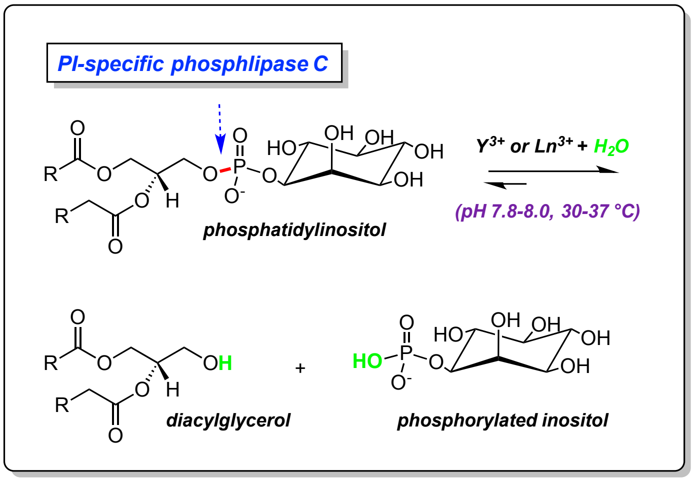

Figure S3. Hydrolytic release of diacylglycerol and phosphorylated inositol observed by Komiyama, Liu, and co-workers upon reacting unactivated phosphatidylinositol (PI) in liposomes and erythrocyte membranes with $CeCl_3$, $EuCl_3$, $LaCl_3$, $TbCl_3$, $TmCl_3$, or YCl_3 (Entries 7 and 6 in **Table S1**).[8, 9] Scissile ester bond is in red. Bond hydrolyzed by PI-specific phospholipase C is identified with a blue arrow.

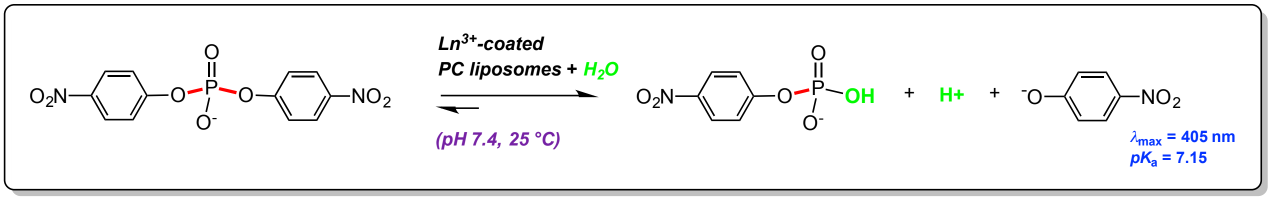

Figure S4. Hydrolytic release of *p*-nitrophenolate anion observed by König and co-workers upon reacting bis-4-nitrophenyl phosphate with Ce(III), Eu(III), La(III), Tb(III), and Yb(III)-coated phosphatidylcholine liposomes (Entry 12 in **Table S1**).[13] Scissile ester bonds are in red.

REFERENCES

1. Boyer, R.F., et al., *2-Hydroxy acids as biochemical ligands. Copper(II)-facilitated hydrolysis of 2-hydroxy acid esters.* J. Inorg. Biochem., 1979. **10**: p. 205-213.
2. Satyarthi, J.K., D. Srinivas, and P. Ratnasamy, *Hydrolysis of vegetable oils and fats to fatty acids over solid acid catalysts.* Appl. Catal. A: Gen., 2011. **391**(1-2): p. 427-435.
3. Ong, L.K., et al., *Direct reuse of Cu-laden wastewater for non-edible oil hydrolysis: Basic mechanism of metal extraction and fatty acid production.* RSC Adv., 2016. **6**(30): p. 25359-25367.
4. Coumans, A.E. and E.J.M. Hensen, *A model compound (methyl oleate, oleic acid, triolein) study of triglycerides hydrodeoxygenation over alumina-supported NiMo sulfide.* Appl. Catal. B: Environ., 2017. **201**: p. 290-301.
5. Moss, R.A., et al., *Lanthanide cleavage of phosphodiester liposomes.* Chem. Commun., 1995(16): p. 1627-1628.
6. Scrimin, P., et al., *Control of permeation of lanthanide ions across phosphate-functionalized liposomal membranes.* J. Am. Chem. Soc., 1998. **120**(6): p. 1179-1185.
7. Scrimin, P., et al., *Ln(III)-catalyzed cleavage of phosphate-functionalized synthetic lipids: Real time monitoring of vesicle decapsulation.* Langmuir 2000. **16**(1): p. 203-209.
8. Matsumura, K. and M. Komiyama, *Hydrolysis of phosphatidylinositol by rare-earth-metal ion as a phospholipase-C mimic.* J. Inorg. Biochem., 1994. **55**(2): p. 153-156.
9. Liu, H.X., et al., *Effects of lanthanide ions on hydrolysis of phosphatidylinositol in human erythrocyte membranes.* Chinese Sci. Bull., 2001. **46**(5): p. 401-403.
10. Kassai, M., R. Teopipithaporn, and K.B. Grant, *Hydrolysis of phosphatidylcholine by cerium(IV) releases significant amounts of choline and inorganic phosphate at lysosomal pH.* J. Inorg. Biochem., 2011. **105**(2): p. 215-223.
11. Cepeda, S.S., D.E. Williams, and K.B. Grant, *Evaluating metal ion salts as acid hydrolase mimics: metal-assisted hydrolysis of phospholipids at lysosomal pH.* Biometals, 2012. **25**(6): p. 1207-1219.
12. Williams, D.E., K. Basnet, and K.B. Grant, *Tuning cerium(IV)-assisted hydrolysis of phosphatidylcholine liposomes under mildly acidic and neutral conditions.* ChemBioChem, 2015. **16**(10): p. 1474-82.
13. Poznik, M., U. Maitra, and B. Konig, *The interface makes a difference: lanthanide ion coated vesicles hydrolyze phosphodiesters.* Org. Biomol. Chem., 2015. **13**(38): p. 9789-9792.
